# Optimization of tissue sampling for *Borrelia burgdorferi* in white-footed mice *(Peromyscus leucopus)*

**DOI:** 10.1371/journal.pone.0226798

**Published:** 2020-01-24

**Authors:** Sonya G. Zawada, Michael E. von Fricken, Thomas A. Weppelmann, Masoumeh Sikaroodi, Patrick M. Gillevet

**Affiliations:** 1 Department of Environmental Science & Public Policy, George Mason University, Fairfax, Virginia, United States of America; 2 Department of Biology, University of Mary Washington, Fredericksburg, Virginia, United States of America; 3 Department of Global and Community Health, George Mason University, Fairfax, Virginia, United States of America; 4 Herbert Wertheim College of Medicine, Florida International University, Miami, Florida, United States of America; 5 Department of Biology, George Mason University, Fairfax, Virginia, United States of America; Tufts University Cummings School of Veterinary Medicine, UNITED STATES

## Abstract

*Peromyscus leucopus* (the white-footed mouse) is a known reservoir of the Lyme disease spirochete *Borrelia burgdorferi*. Sampling of white-footed mice allows for year-round *B*. *burgdorferi* surveillance as well as opportunities to establish the diversity of the different variants in a geographic region. This study explores the prevalence of *B*. *burgdorferi* infections in the tissues of white-footed mice, investigates the correlations between *B*. *burgdorferi* infected tissues, and determines the optimum field methods for surveillance of *B*. *burgdorferi* in *P*. *leucopus*. A total of 90 mice and 573 tissues (spleen, liver, ear, tongue, tail, heart, and kidney) were screened via nested PCR for *B*. *burgdorferi* infections. A large number of infections were found in the 90 mice as well as multiple infections within individual mice. Infections in a single mouse tissue (spleen, liver, ear, tongue and tail) were predictive of concurrent infection in other tissues of the same mouse at a statistically significant level. Ear tissue accounted for 68.4% of detected infections, which increased to 78.9% of the infected mice with the inclusion of tail samples. The use of ear punch or tail snip samples (used individually or in tandem) have multiple advantages over current Lyme disease ecological studies and surveillance methodologies, including lower associated costs, minimization of delays, year-round *B*. *burgdorferi* testing opportunities, as well as longitudinal monitoring of *B*. *burgdorferi* in defined geographic regions. In the absence of an effective vaccine, personal prevention measures are currently the most effective way to reduce Lyme disease transmission to humans. Thus, the identification and monitoring of environmental reservoirs to inform at-risk populations remains a priority. The sampling methods proposed in this study provide a reasonable estimate of *B*. *burgdorferi* in white-footed mice in a timely and cost-effective manner.

## Introduction

Lyme disease is the most common tick-borne illness in the United States, with evidence that both the prevalence and geographic range are increasing [1–3]. Infections are caused by spirochete bacteria of the genus *B*. *burgdorferi* and transmitted by ticks of the genus *Ixodes* to both animals and humans. In the mid-Atlantic region, where Lyme disease is most prevalent, the white-footed mouse (*Peromyscus leucopus* Rafinesque) is the primary reservoir of *B*. *burgdorferi* [4]. Human infections typically present with a transient rash at the site of the tick bite and may progress to involve the joints, nervous system, and heart; resulting in lingering sequelae such as arthritis, neuropathy, or potentially fatal cardiac disease [5]. Without an approved human vaccine in the United States, prevention of human infections relies primarily on reducing exposure to ticks [6].

With the realization that the prevalence of Lyme disease has likely been greatly underestimated, research and development of surveillance techniques to monitor for environmental reservoirs has become a cornerstone of public health interventions [7]. Traditional environmental sampling strategies involve trapping, dissecting, and homogenizing tissue and organs from mice for PCR screening of *B*. *burgdorferi* [8]. The use of PCR detection in non-lethal ear punch biopsies has been reported to have an increased rate of detection, however, due to tissue tropisms of different genetic variants, it remains unclear if ear punches are an accurate indicator of the true infection prevalence [9]. In an effort to determine the most efficient sampling methods and the agreement between ear punches and other organs, this study examined the prevalence of *B*. *burgdorferi* in multiple tissues of wild-caught *P*. *leucopus* in an endemic region of Fairfax County, Virginia.

## Material and methods

### Field collections

Mice were collected in Fairfax County, Virginia utilizing Sherman® live steel traps in spring/summer/fall seasons and museum special traps in winter (December-March). Between forty and fifty traps were set along brush piles bordering fields with a bait mixture of peanut butter and oatmeal at dusk, marked with flagging tape, and GPS-tagged. Collected mice were retrieved the following morning between the hours of 06:00 and 07:00, documented, given a field identification number, wrapped in a piece of newspaper, and brought back to the laboratory for tissue harvest and sample processing. Specimens collected via Sherman® live steel traps were dispatched in a CO_2_ chamber and those collected via museum special traps were dispatched via vertebral dislocation upon triggering the trap mechanism.

Geographic localities for trapping sites are as follows: I-66 Transfer Station (38.851443 -77.380081), Goodwood (38.834570 -77.358750), Huntley Meadows Park (38.756417 -77.115347), Graves (38.771210 -77.095720), Stoneybrooke (38.770943 -77.096315).

### Laboratory analysis

Collected mice were necropsied to separate spleen, liver, ear, tongue, tail, heart, and kidney tissues prior to storage in separate sterile microcentrifuge tubes at -80°C. DNA extraction was performed using the Qiagen DNeasy Blood & Tissue Kit according to the manufacturer’s instructions, diluted (1:5 in DEPC water), and both original DNA and dilutions were stored at -80°C for future use. Detection of the outer surface protein C (*OspC*) of *B*. *burgdorferi* was performed on mouse tissues’ using a nested PCR protocol as previously described [10].

### Ethics statement

An IACUC was completed and approved by George Mason University under the Institutional Biosafety Committee (IBC) protocol# 12-28Mod1. Trapping was conducted under the Virginia Department of Game and Inland Fisheries (VADGIF) permit number 032199, 035522, 046610, and 050364.

### Statistics

**Fig 1:**
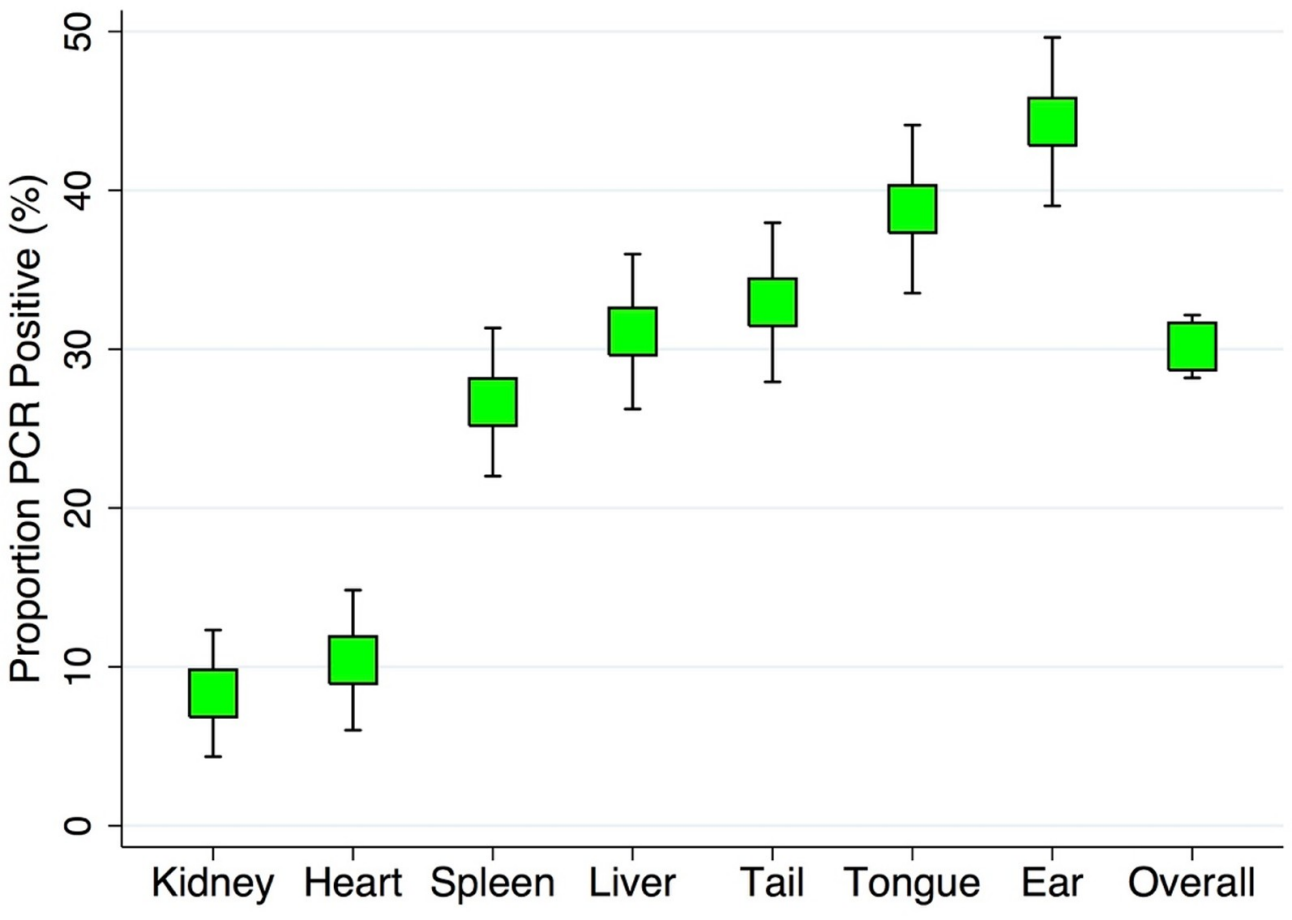
A graphic representation of the proportion of samples with *B*. *burgdorferi* DNA detected by PCR by tissue type with 95% confidence intervals.

**Fig 2:**
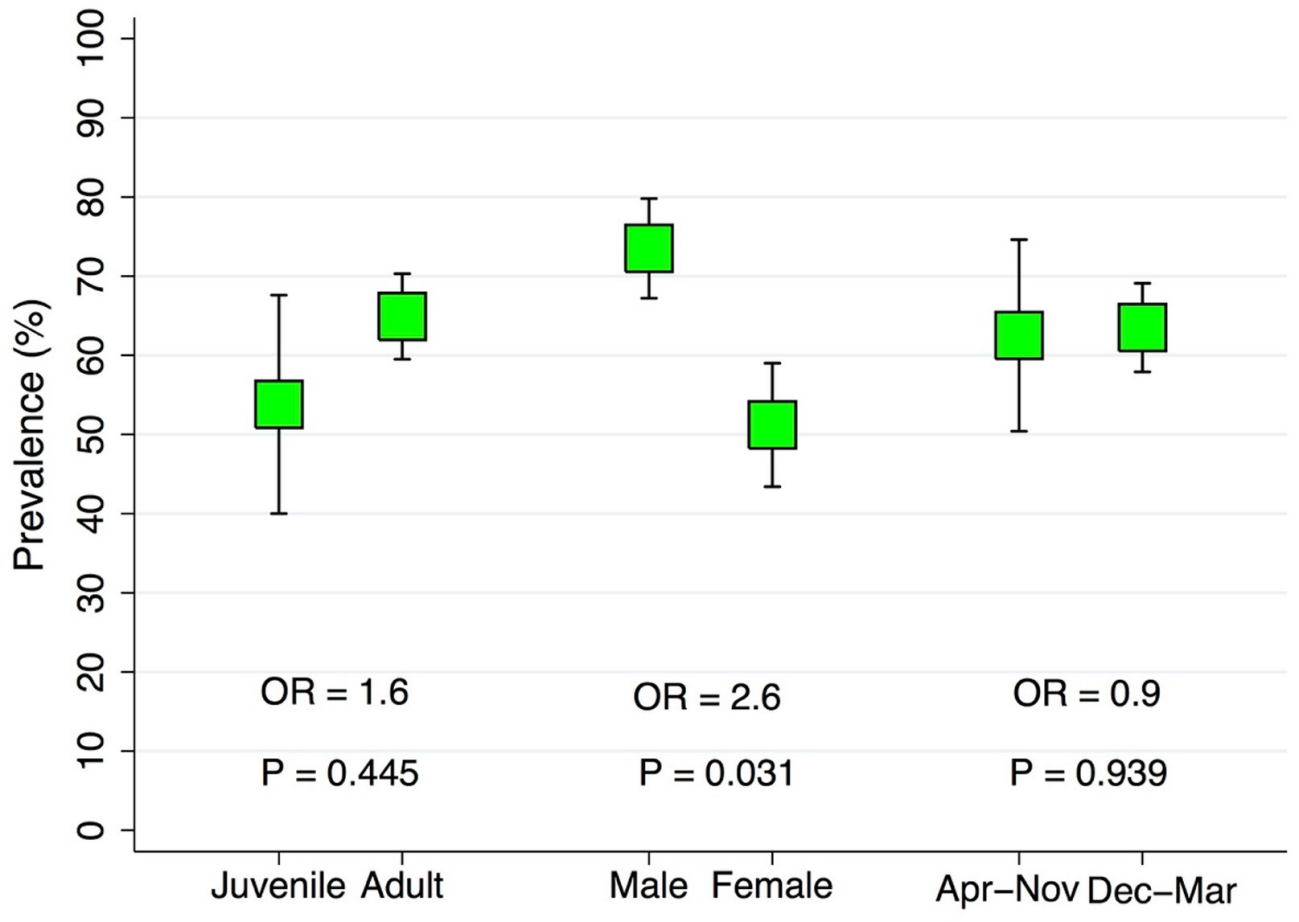
A graphic representation of the proportion of mice with *B*. *burgdorferi* DNA detected by PCR by age, sex, and trapping season with 95% confidence intervals and corresponding significance value (P) and odds ratios (OR) from comparison with categorical logistic regression models.

A total of 90 mice and 537 tissues samples were successfully collected and screened using the above methods. A mouse was considered positive if any of the tissues had detectable *B*. *burgdorferi* DNA. The detection prevalence by tissue was calculated as the total number of positive tissue samples divided by the number of tissue samples, overall and for each type of tissue. These results were displayed in tabular form and graphically (Table 1 and Fig 1) with 95% confidence intervals for the proportion of positive samples from each tissue type. A logistic regression model was used to compare the likelihood of a tissue containing detectable *B*. *burgdorferi* DNA compared to all other tissues and expressed as an odds ratio with 95% confidence intervals. The sensitivity of using and individual tissue type to detect an infected mouse was calculated by dividing the number of positive tissue samples from each tissue type among positive mice divided by the total number of positive mice an expressed as a percentage. The proportion of mice that were positive by age (juvenile or adult), sex (male or female), and season (April to November or December to March) was tabulated and displayed graphically with 95% confidence intervals (Fig 2). Logistic regression models were used to compare the likelihood of being infected by age, sex, or season, with and without adjustment and expressed as odds ratios with 95% confidence intervals.

**Table 1:** Tabulation of samples with detectable *B*. *burgdorferi* DNA by tissue type.

Tissue	(n)	Pos.	% Pos.	SE	95% Conf. Int.	
**Spleen**	90	24	26.7	4.7	17.5	35.8
**Liver**	90	28	31.1	4.9	21.5	40.7
**Ear**	88	39	44.3	5.3	33.9	54.7
**Tongue**	85	33	38.8	5.3	28.5	49.2
**Tail**	88	29	33.0	5.0	23.1	42.8
**Heart**	48	5	10.4	4.4	1.8	19.1
**Kidney**	48	4	8.3	4.0	0.5	16.2
**Total**	537	162	30.2	2.0	26.3	34.0

The number of tissue samples (n), the number of positive samples (Pos.), the percentage of positive samples (% Pos.) are presented along with standard error and 95% confidence intervals for the proportion of positive samples by tissue type and for all tissue samples (Total).

## Results

A summary of the number of positive tissue samples, number of total tissue samples, and the proportion of samples from each tissue type with detectable *B*. *burgdorferi* are presented in Table 1. Of the 90 mice screened *B*. *burgdorferi*, 63.3% (57/90) of the mice were positive. The prevalence by age, sex, and season trapped appear in Fig 1.

Male mice had higher frequency of infection than females (73.5% vs 51.2%) and were 2.6 times more likely to be infected (95% CI OR: 1.09, 6.37; P= 0.031). Adult mice had higher prevalence than juveniles (64.9% vs 53.6%), but were not more likely to be infected than adults (P =0.445). Mice trapped in April to November had a similar prevalence compared to those trapped in December to March (62.5% vs 63.5%), and were not significantly more likely to be infected (P= 0.939). After adjustment for age, sex, and season trapped, sex remained significant with a similar estimated odds of infection (P= 0.041; OR = 2.5).

Of the 537 tissues samples screened for *B*. *burgdorferi*, 30.4% (163/537) of the tissue samples were positive. The prevalence of positive samples by tissue type is presented in Fig 2.

The most commonly infected tissues were ear (44.3%), tongue (38.8%), tail (33.0%), liver (31.1%), spleen (26.7%), heart (10.4%), and kidney (8.3%). Of infected mice, the sensitivity of the following tissues as an individual sample to identify an infected mouse were as follows: ear (69.6%), tongue (62.3%), tail (51.8%), liver (49.1%), spleen (43.9%), kidney (13.8%), and heart (8.8%). On average, infected mice had 2.8 tissues types with detectable *B*. *burgdorferi*, with 47 (82.5%) positive in at least two tissue types, 30 (52.6%) positive in at least three tissue types, 16 (28.1%) positive in at least four tissue types, and 13 (22.8%) positive in five or more tissue types. Compared to all other tissues, only the ear, kidney, and heart had significantly different proportions of positive samples. The ear was 2.1 times more likely to be positive (P= 0.002; 95% CI OR: 1.32, 3.37). The kidney was 81% less likely to be positive (P= 0.002; 95% CI OR: 10.063, 0.539). The heart was 75% less likely to be positive (P= 0.004; 95% CI OR: 0.095, 0.638). Compared to all other tissues the odds of each tissue to be infected was as follows: tongue (OR: 1.59; P= 0.059), tail (OR: 1.17; P= 0.534), liver (OR: 1.05; P= 0.831), and spleen (OR: 0.81; P= 0.428).

## Discussion

In this study it was observed that the presence of *B*. *burgdorferi* in tissues of infected mice can be highly variable. Of note, it was discovered that ear tissue accounted for 68.4% of the overall prevalence, which increased to 78.9% of the infected mice with the inclusion of tail samples. Additionally, clear differences in likelihood of testing positive between tissues were observed when comparing the ear, heart, or kidney to all other tissues, with ear tissue 2.1 times more likely to be positive (P = 0.002).

In the meantime, the use of ear punch or tail snip samples (used individually or in tandem) have multiple advantages over current Lyme disease surveillance methodologies [11]. First, the resources involved in whole mouse collection, necropsy, and dissection are a limiting factor in surveillance; ear punches or tail snips could be analyzed at a much lower cost and with minimal delay to provide real time results. Secondly, the use of ear punches or tail snips is non-lethal and makes longitudinal monitoring of *B*. *burgdorferi* possible as rodents may be tagged and trapped on multiple occasions. Lastly, the use of white-footed mice in addition to tick sampling allows for year-round surveillance of *B*. *burgdorferi* that would otherwise be difficult in colder months when ticks activity is greatly reduced [12]. Although the ear had the highest prevalence of any tissue sampled, and the tail had the third highest, the use of only these two tissues would have under estimated the prevalence of infection in this population by 20%. This could be remedied by conducting a full mouse tissue survey with comparison to ear and tail punches when establishing an ecological study focused on transmission cycles in nature, or a surveillance program for a specific geographic region in order to account for underestimation.

In the absence of an effective vaccine, personal prevention measures are currently the most effective way to reduce Lyme disease transmission to humans [13]. Thus, the identification and monitoring of environmental reservoirs to inform at-risk populations remains a priority. The sampling methods proposed in this study provide a reasonable estimate of *B*. *burgdorferi* in white-footed mice in a timely and cost-effective manner relative to whole animal necropsy. We propose that the methods outlined in this paper could be applied to longitudinal studies of infection in white-footed mice, the monitoring of *B*. *burgdorferi* variants, and determining the rate of clearance of previously acquired *B*. *burgdorferi* variants in a natural setting.
